# The Nuclear Receptors of *Biomphalaria glabrata* and *Lottia gigantea*: Implications for Developing New Model Organisms

**DOI:** 10.1371/journal.pone.0121259

**Published:** 2015-04-07

**Authors:** Satwant Kaur, Susan Jobling, Catherine S. Jones, Leslie R. Noble, Edwin J. Routledge, Anne E. Lockyer

**Affiliations:** 1 Institute of Environment, Health and Societies, Brunel University London, Uxbridge, United Kingdom; 2 School of Biological Sciences, University of Aberdeen, Aberdeen, United Kingdom; Gettysburg College, UNITED STATES

## Abstract

Nuclear receptors (NRs) are transcription regulators involved in an array of diverse physiological functions including key roles in endocrine and metabolic function. The aim of this study was to identify nuclear receptors in the fully sequenced genome of the gastropod snail, *Biomphalaria glabrata*, intermediate host for *Schistosoma mansoni* and compare these to known vertebrate NRs, with a view to assessing the snail's potential as a invertebrate model organism for endocrine function, both as a prospective new test organism and to elucidate the fundamental genetic and mechanistic causes of disease. For comparative purposes, the genome of a second gastropod, the owl limpet, *Lottia gigantea* was also investigated for nuclear receptors. Thirty-nine and thirty-three putative NRs were identified from the *B. glabrata* and *L. gigantea* genomes respectively, based on the presence of a conserved DNA-binding domain and/or ligand-binding domain. Nuclear receptor transcript expression was confirmed and sequences were subjected to a comparative phylogenetic analysis, which demonstrated that these molluscs have representatives of all the major NR subfamilies (1-6). Many of the identified NRs are conserved between vertebrates and invertebrates, however differences exist, most notably, the absence of receptors of Group 3C, which includes some of the vertebrate endocrine hormone targets. The mollusc genomes also contain NR homologues that are present in insects and nematodes but not in vertebrates, such as Group 1J (HR48/DAF12/HR96). The identification of many shared receptors between humans and molluscs indicates the potential for molluscs as model organisms; however the absence of several steroid hormone receptors indicates snail endocrine systems are fundamentally different.

## Introduction

The tropical freshwater snail *Biomphalaria glabrata* is an intermediate host for several digenean trematode parasitic worms, including *Schistosoma mansoni*, the causative agent of human intestinal schistosomiasis. Human schistosomiasis is the most widespread trematode infection affecting around 200 million people, leading to a chronic debilitating disease and up to 200,000 deaths per year, across 75 developing countries [1]. Due to its medical significance as an intermediate host, *B*. *glabrata* has been the focus for much research, including several gene discovery projects [2–4]. Tools have been developed for investigating genomic and transcriptomic attributes of this species, including a BAC library for genome sequencing [5]; a 5K cDNA microarray [6]; a 1.2K oligo microarray [7] and the means to selectively silence gene expression in the snail (RNAi: [8,9]). This background of research has culminated in the sequencing the *B*. *glabrata* genome [10] (http://129.24.144.93/blast_bg/2index.html). The progress made in developing these resources has also provided the potential for the snail to become a new model organism for other purposes, including testing and identification of endocrine disrupting chemicals (EDCs) and for understanding fundamental biology, including endocrinology.

Endocrine and metabolic disease are among the most common contemporary human afflictions, the prevalence of which has been well defined in large population-based studies (for example, [11,12]). Some of the causes of these are not immediately obvious, but may be related to increasing exposure to EDCs [13]. As a consequence, not only is more testing of potential EDCs needed, but a better understanding of endocrine function and disruption is also required. A solution that is appropriate and in keeping with the three R’s (replacement, refinement and reduction) ethos of animal research [14], is to exploit the use of invertebrate organisms which may offer a simplified model for research, as well as providing a faster, cheaper and more ethically acceptable alternative to mammalian testing, at least during initial chemical screening [15]. As NRs play key roles in endocrine and metabolic functions [16], cross-species comparative studies of the conservation of these genes within invertebrate genomes may identify new model systems for the testing of chemicals with endocrine disrupting potential without using vertebrates. In addition to this, *B*. *glabrata* has recently been proposed specifically for developmental toxicity, acute toxicity and mutagenicity testing in order to establish standardised protocols to assess environmental risks [17]. Therefore a better knowledge of normal endocrine function in molluscs will enable us to understand the full impact of EDCs in the environment, where they have been shown to affect both vertebrate and invertebrate species [18].

One class of transcription factor involved in regulating endocrine function in vertebrates are the nuclear receptors (NRs). NRs regulate and coordinate multiple processes by integrating internal and external signals, thereby maintaining homeostasis (reviewed in [16,19]) These proteins exhibit strong similarities in their mode of action due to their conserved molecular structure, which includes a DNA-binding domain (DBD), consisting of two Cys4 zinc fingers, and the ligand-binding domain (LBD), which not only controls signalling by binding small lipophilic molecules, called ligands, but also binds co-activators and co-repressors [20]. Both the LBD and D box domain of the DBD mediate receptor hetero- or homo-dimerization (reviewed in [21]). Biochemical studies and crystal structure of the LBD reveal that ligand binding triggers a conformational change, causing bound co-repressors to be displaced by co-activators, leading to gene expression [22]. NRs are classified into six distinct families by sequence homology using a phylogenetic approach [23]. Based on their conserved nature and their biologically essential roles throughout the Metazoa, NRs are believed to have emerged early in animal evolution, prior to the bilaterian ancestor [24–26].

Molluscs are affected by EDCs and one of the most cited examples is that of the marine pollutant tributyltin (TBT), found in antifouling paint, which is responsible for imposex in at least 195 species of gastropods worldwide (reviewed in [27,28]). TBT has been shown to act through binding to an NR, the retinoid X receptor (RXR) [29,30]. Other environmental pollutants could act through the estrogen receptor (ER) or androgen receptor (AR) and there is also some evidence of a role for androgen and estrogen-like molecules in the reproductive cycle of molluscs [31,32]. ER orthologues have previously been reported in molluscs (eg [33–37]) although their function is currently unconfirmed [38,36,39,37,40], since, for example, the identified ER homologue from the oyster *Crassostrea gigas* is unresponsive to estrogen [37], The presence of an ER in molluscs and the potential to respond to estrogens is confounded by the absence of aromatase (CYP19) [41], the enzyme required for the conversion of testosterone to estradiol, but it is possible that another enzyme catalyses the aromatase reaction [38]. The presence of ARs in molluscs has been inferred [32,42], but this remains an area of controversy [43] since homologues have not been identified despite investigations specifically searching for the gene [44]. A recent survey of NRs in *C*. *gigas* also did not identify an AR [45].

Previously only the RXR has been characterised in *B*. *glabrata* [46]. With the recent availability of a draft genome for *B*. *glabrata* it is particularly timely to search for the NRs of *B*. *glabrata*. The genome of the owl limpet, *L*. *gigantea* [47], a marine gastropod, for which 26 NRs have already been identified [25,48,49], was compared with the snail NR repertoire. These species, from different families, represent distinct gastropod lineages. The sequences of identified putative genes were confirmed using transcript data from the *L*. *gigantea* genome portal and by amplification of products from *B*. *glabrata* mRNA. We assess the conservation, divergence, and uniqueness of gastropod NRs in comparison with the previously characterised receptors of a vertebrate (*Homo sapiens*), insect (*Drosophila melanogaster*), nematode worm (*Caenorhabditis elegans*) (reviewed in [50]) and the parasitic trematode (*S*. *mansoni*) (reviewed in [51]).

## Methods

### Identification of nuclear receptors from *B*. *glabrata* and *L*. *gigantea* genomes

Human AR (GenBank: EAX05380) and ERα (GenBank: AAI28575) gene sequences were used to search for expressed sequence tags (ESTs) in *B*. *glabrata* and *L*. *gigantea* at National Centre for Biotechnology Information (NCBI) using TBlastX with default parameters [52]. These ESTs were then used to search for homologues at the preliminary *B*. *glabrata* genome (version 4.01: http://129.24.144.93/blast_bg/2index.html) (TBlastX expect limit:1e-04) and assembled *L*. *gigantea* transcripts from the filtered gene models in the JGI Genome Portal (http://genome.jgi-psf.org/Lotgi1/Lotgi1.info.html) using BLASTN (expect limit: 1e-99). This was followed by a wider search using NR gene sequences spanning all the groups present in humans and *D*. *melanogaster*. A secondary search using the identified *L*. *gigantea* and *B*. *glabrata* NR genes against both the gastropod genomes using a low expect threshold value identified additional family members, some of which were distinct from human and fly NRs. Query sequences were filtered for low complexity regions. Identified contigs from *B*. *glabrata* were downloaded and processed through GENSCAN [53] (http://genes.mit.edu/GENSCAN.html) using default parameters, to identify predicted coding regions, intron-exon boundaries, and peptides. Predicted peptides were assessed using BLASTP (expect limit: 1e-10) against the non-redundant GenBank database to check for the presence of the NR domains (LBD/DBD). The identified nucleotide sequences from both molluscs containing NR domains were analysed for redundancy using clustering analysis with sequence overlap cut-off set at 0.5 and segment coverage cut-off at 0.25 in Seqtools (8.4ver) (http://www.seqtools.dk/).

The identified genes were named based on the phylogenetic analysis and sequence similarity of the full-length sequence to previously characterized human and *D*. *melanogaster* NRs according to the recognised nomenclature [23]. Small sequence fragments of putative NRs from *B*. *glabrata* too short to be classified were left out of the subsequent analysis. Where there was no vertebrate or invertebrate homologue, subfamily classification was made, with a number representing the subfamily, a capital letter for the group, and a number for the individual gene.

### RNA isolation and cDNA synthesis

Total RNA was isolated from whole homogenized adult *B*. *glabrata* snails using TRI-reagent (Sigma-Aldrich, St Louis, USA) according to the manufacturer’s protocol and treated with DNAse to eliminate contaminating genomic DNA. Total RNA was extracted from embryonic samples using the RNeasy Fibrous tissue mini kit (Qiagen, Limberg, Netherlands), which included a DNAse treatment as part of the protocol. Quantification and purity of each RNA sample were determined by spectrophotometry (Nanodrop, Thermo Fisher Scientific Ltd. Waltham, USA), and the RNA integrity was visually checked by agarose gel electrophoresis. 4μg of total RNA from adult snails and 1.5μg total RNA from embryonic samples was reverse transcribed in a 20μl reaction using the Superscript III cDNA synthesis kit (Invitrogen, Life Technologies, Carlsbad. USA) with 5 μM of a custom oligo (dTAP) primer (TGACTCGAGTCGACATCGAT_20_) following the manufacturer’s instructions. Residual RNA was removed by adding 1μl of RNase H (2 U/μl) to the reaction and incubating it at 37°C for 20 min. RT-PCR with 18S primers (18S-F: CGCCCGTCGCTACTATCG and 18S-R: ACGCCAGACCGAGACCAA) [54] verified successful cDNA synthesis.

### Polymerase chain reaction

Specific PCR primers (S1 Table) were designed using PRIMER3 (version 0.4.0: http://bioinfo.ut.ee/primer3-0.4.0/), to amplify fragments from DBD and/or LBD for each *B*. *glabrata* NR to confirm transcription and sequence. 25μl PCRs contained 2μl cDNA (diluted 1 in 20), 1 X PCR Buffer, 2.5mM MgCl_2_, 0.2mM dNTPs, 0.5μM forward and reverse primers and 1.25U *Taq* DNA polymerase (Bioline, London, UK). Reaction conditions were 2 min at 95°C followed by 35 cycles of 30 sec at 95°C, 30 sec at 55–64°C (optimised for each primer pair (S1 Table)) 1 min 30 sec at 72°C, with a final extension of 5 min at 72°C. Amplified products were analysed by gel electrophoresis and products of an appropriate size were gel extracted where necessary and sequenced (Sequencing facility, Wolfson Wellcome Biomedical Laboratory, Natural History Museum, UK). Sequences were deposited in GenBank Accession Nos: JZ390894-JZ390939

### Sequence alignments and phylogenetic analysis

The sequenced NR transcripts from *B*. *glabrata* were translated using proteomics tools at EXPASY (http://www.expasy.org/). The predicted peptide sequences from both *B*. *glabrata* and *L*. *gigantea* were analysed using PFAM domain analysis [55] (PF00104 and PF00105) and PANTHER [56], a hidden Markov model-based method (PTHR24082) confirming the NR domains. The NCBI program Simple Modular Architecture Research Tool (SMART, [57]) was used for the identification of DNA-binding domain (DBD) and ligand binding domain (LBD) regions which were then aligned with the DBD and LBD regions of human (*H*. *sapiens*), fruit fly (*D*. *melanogaster*) nematode (*C*. *elegans*) and parasitic trematode (*S*. *mansoni*) NRs (Table 1). The NR domains were aligned using default parameters in ClustalX and converted to Nexus format using default parameters in Mesquite v2.75. The DBD and LBD from NRs of the other species were obtained using the conserved domain database (CDD) and reconfirmed using SMART. The NR2A subfamily expansion that contains a large number of the *C*. *elegans* NRs [58] was disregarded and only 15 NRs from *C*. *elegans* that are broadly conserved among animal phyla were included in this study for comparative analysis.

**Table 1:** Sequence identification of all nuclear receptors for species in the study: NRs from fly, human, nematode and trematode (NCBI accession numbers), compared to identified *L*. *gigantea* NRs (protein identification numbers: JGI genome portal version 1.1) and *B*. *glabrata* NRs (Contig numbers: Preliminary Bg Genomic Data (version 4.01)).

Group	*C*. *elegans*	Accession	*S*. *mansoni*	Accession	*D*. *melanogaster*	Accession	*B*. *glabrata*	Contig	*L*. *gigantea*	Protein ID	*H*. *sapiens*	Accession
**0A**					KNR	CAA31709						
					KNRL	AAF51627						
					EGON	CAA34626						
**0B**							BgDAX	182	LgDAX	153776	DAX1	AAH11564
											SHP	AAH30207
**1A**			THRa	AAR32912			BgTHR^‡^	3.1	LgTHR	207623	THRα	AAH08851
			THRb	AAR29359							THRβ	AAI06931
**1B**	ODR7	AAC46497	RAR-like*	CCD76558			BgRAR	398	LgRAR	207867	RARα	AAH08727
											RARβ	AAH60794
											RARγ	AAA63254
**1C**							BgPPAR1	2052	LgPPAR1	174409	PPARα	AAB32649
							BgPPAR2	1275	LgPPAR2	238472	PPARβ	AAA36469
											PPARγ	AAH06811
**1D**	HR85	AAO39185			E75	AAN11687	BgE75	29	LgE75	136477	Rev-erb-a	AAH56148
							BgRev-erb	160	LgRev-erb	168854	Rev-erb-b	AAH45613
							BgNR1D1^+^ *	1939				
							BgNR1D2^+^ *	1958				
							BgNR1D3^+^ *	201				
**1E**			E78	AAR30507	E78	AAF51692	BgE78	534	LgE78	163301		
**1F**	HR23	P41828			HR3	AAA28461	BgHR3	73	LgHR3	167096	RORα	AAH08831
							BgROR	74	LgROR	155536	RORβ	AAH93774
											RORγ	AAA64751
**1G**	HR14	AAA96982										
**1H**					EcR	NP724456	BgEcR	1481	LgEcR	170342	LXRα	AAV38218
											LXRβ	NP_009052
											FXR	AAI30574
**1I**											VDR	AAB95155
											PXR	AAD05436
											CAR	AAY56401
**1J**	DAF12	AAD34462	HR96α(CAR) *	AAV80235	HR96	AAC46928	BgNR1J1	48	LgNR1J1	163618		
	HR8	AAP31437	HR96	AAW88541			BgNR1J2	954	LgNR1J2	63892		
	HR48	CAD36502					BgNR1J3	2148	LgNR1J3	163956		
							BgNR1J4	3233				
**1K**	HR1	AAC48174										
**2A**	HR49	CAD57702	HNF4	CCD80248	HNF4	AAF52702	BgHNF4	2702	LgHNF4	73247	HNF4	NP_849180
											HNF4γ	NP_004124
**2B**			RXR1	CCD59553	USP	AAF45707	BgRXR	3695	LgRXR	206562	RXRα	AAI10999
			RXR2	AAD33428							RXRβ	AAC18599
											RXRγ	AAA80681
**2C/D**	HR41	NP_500073	TR	AAV80236	HR78	Q24142	BgTR	2718	LgTR*	231979	TR2	AAA36761
											TR4	NP_003289
**2E**	FAX	AAD55066	TLL*	AAW88549	TLL	AAB71371	BgTLX*	3	LgTLX	130367	TLX	AAL05871
	HR67	CAA97428	PNR*	CCD82728	PNR	AAF58145	BgDSF	42	LgDSF	64143	PNR	AAD28301
			DSF*	AAW88537	DSF	AAF52303	BgFAX1	148	LgFAX1	168960		
					FAX1	AAF54133	BgPNR^‡^	3827	LgNR2E	232946		
									LgPNR	171552		
**2F**	UNC55	CAP16277	COUP-TFI*	CCD77973	SVP	NP_524325	BgCOUP-TFa	5703	LgCOUP-TFa	BgCOUP-TFa	COUP-TFα	AAH04154
			COUP-TFII*	AAW88536							COUP-TFβ	AAH42897
											EAR2	AAH02669
**3A**							BgER	469	LgER	132166	ERα	AAI28575
											ERβ	AAV31779
**3B**					ERR	AAL37554	BgERR	751	LgERR	168715	ERRα	AAH63795
											ERRβ	AAC99409
											ERRγ	EAW93335
**3C**											GR	AAH15610
											MR	AAA59571
											PR	AAA60081
											AR	EAX05380
**4A**	HR6	CAA85271	NR4A5*	AAR28090	DHR38	AAF53914	BgHR38	27	LgHR38	94341	NGFIB	CAG32985
							BgNR4a	2842	LgNR4a	182258	NURR1	AAH09288
											NOR1	CAI95138
**5A**	HR25	CAA91028	FTZ-F1α (SF1)*	CCD77387	FTZ-F1	AAN11667	BgFTZ-F1	1318	LgFTZ-F1	196916	SF1	AAH32501
											LRH1	AAI18572
**5B**			HR39(FTZ-F1)	CCD77680	DHR39	AAN11107	BgHR39	3667	LgHR39	94029		
**6A**	HR91	CAB60329			DHR4	AAX73355	BgHR4	1866	LgHR4a	128948	GCNF	AAB96828
									LgHR4b	162354		
**2DBDNR**			2DBDα*	AAW88533			Bg2DBDNR1*	304	Lg2DBDNR1*	168696		
			2DBDβ*	AAW88534			Bg2DBDNR2* ^‡^	1296	Lg2DBDNR2*			
			2DBDγ*	AAW88550								
**Unclassified**							BgNRU1	221				
							BgNRU2	293				
							BgNRU3	180				
							BgNRU4* ^‡^	250				

*NRs not used in phylogenetic tree;

^+^ NRs are short fragments that could be multiple partial genes or parts of a full transcript;

^‡^ NRs not found expressed in *B*. *glabrata*

Phylogenetic reconstruction was performed using Maximum Likelihood analysis with Bayesian inference and MEGA 6.06. Bayesian inference was conducted with MrBayes version 3.1.2 [59] using the WAG model; the best fitting substitution model determined by both Akaike information criterion and Bayesian information criterion frameworks (ProtTest (v1.4) [60]). Two independent runs of Markov Chain Monte Carlo (MCMC) analysis were performed, with four chains run for 7 million generations with the ‘temperature’ parameter at 0.10, prior probabilities with default values and sampling every 2000 generations. The first 2 million generations were discarded as burn-in because the Log likelihood values were plotted and found to be asymptotic well before the burn-in fraction. Convergence between the independent MCMC runs was examined by the average deviation of the split frequencies and the potential scale-reduction factor (PSRF), which was 1.00. Clades with posterior probabilities >95% were considered well supported. All PSRF values for MrBayes analyses were 1.00. For Maximun Likelihood analysis, the Jones-Taylor-Thornton (JTT) substitution model [61] was used, with a gamma distribution of rates between sites (eight categories, parameter alpha, estimated by the program). Support was evaluated by 1000 bootstrap replicates.

Maximum parsimony was also used to provide additional phylogenetic support for classification, naming, and the phylogenetic relationships observed between *B*. *glabrata*, *L*. *gigantea* and nuclear receptors from selected species. Maximum parsimony with heuristic searches, branch swapping set to tree-bisection-reconnection, topological constraints not enforced, and 1000 bootstrap replicates were performed using MEGA6.06 [62]. An appropriate out-group to root the sequences was difficult for such a diverse and ancient nuclear receptor family and so the phylogenies were mid-point rooted using Dendroscope (ver3.2.10) [63].

## Results and Discussion

### Nuclear receptor genes

Thirty nine NR sequences were identified from the genome of *B*. *glabrata* and a total of 33 from *L*. *gigantea* consisting of 7 newly identified NRs and confirming 26 sequences which had been previously identified from *L*. *gigantea* in previous studies [25,48,49] (Table 1). An exhaustive search with mammalian and *D*. *melanogaster* NRs and with the identified mollusc NRs found no further NR homologs. Representative genes for each of the major nuclear receptor groups were detected, suggesting the assembled genome for *B*. *glabrata* provides a good representation of total gene content with physical genome coverage of approximately 27.5X (Genbank: APKA00000000.1). The numbers found are comparable to the 48 NRs reported in the human genome [64], 49 in mice [65] and 47 in rats [66]. Insects have lower numbers; only 21 NR genes are found in *D*. *melanogaster* [67], 22 in *Apis mellifera* [68], and 21 in *Tribolium castaneum* [69]. Over 270 NRs have been found in *C*. *elegans* [70] but only 21 NRs in the trematode *S*. *mansoni* [71]. The unusually large number of NRs in *C*. *elegans* is due to extensive proliferation of one gene (HNF4) within the nematode phylum [58]. 43 NRs were recently identified from the Pacific oyster, *C*. *gigas*, a marine bivalve, [45], similar numbers to those we identified from the gastropods.

### Expression of *B*. *glabrata* nuclear receptor mRNAs

Each predicted nuclear receptor was evaluated for expression in *B*. *glabrata* adult snails, by amplifying cDNA fragments with receptor-specific primers, to demonstrate that the predicted genes were expressed as transcripts and confirm their sequence. Primer pairs for which fragments were not obtained from adults were also tested on embryos. Expression was confirmed for 34 out of 39 identified receptor genes (Table 1), 31 from cDNA derived from whole adult snails, while 3 nuclear receptors, BgTLX, BgDSF and BgFAX1, were not expressed in adult snails but were identified in 96hrs/120hrs embryos. These receptors belong to NR2E sub family and may be involved in embryonic development as reported in *Daphnia pulex* [72]. All amplicons corresponded to the predicted sequence length and sequencing confirmed the predicted identity of the gene products (GenBank Accession Nos: JZ390894-JZ390939). For *L*. *gigantea*, *in silico* searches identified sequenced transcripts for 21 out of 33 NR genes (S2 Table). Three *B*. *glabrata* putative NRs (BgNR1D1/2/3) for which only partial sequences were obtained from the genome were not included for further phylogenetic analysis, since it was not possible to ascertain if they are multiple partial genes or parts of the same transcript. In addition to this BgTLX, BgNRU4 and LgTR were not analysed further as these were also incomplete sequences. A separate analysis was made of 2DBD genes from *B*. *glabrata* and *L*. *gigantea* with other previously identified 2DBD sequences from *S*. *mansoni* and other species as the 2DBD region could not be aligned with the other NRs.

### Phylogenetic analyses

**Fig 1:**
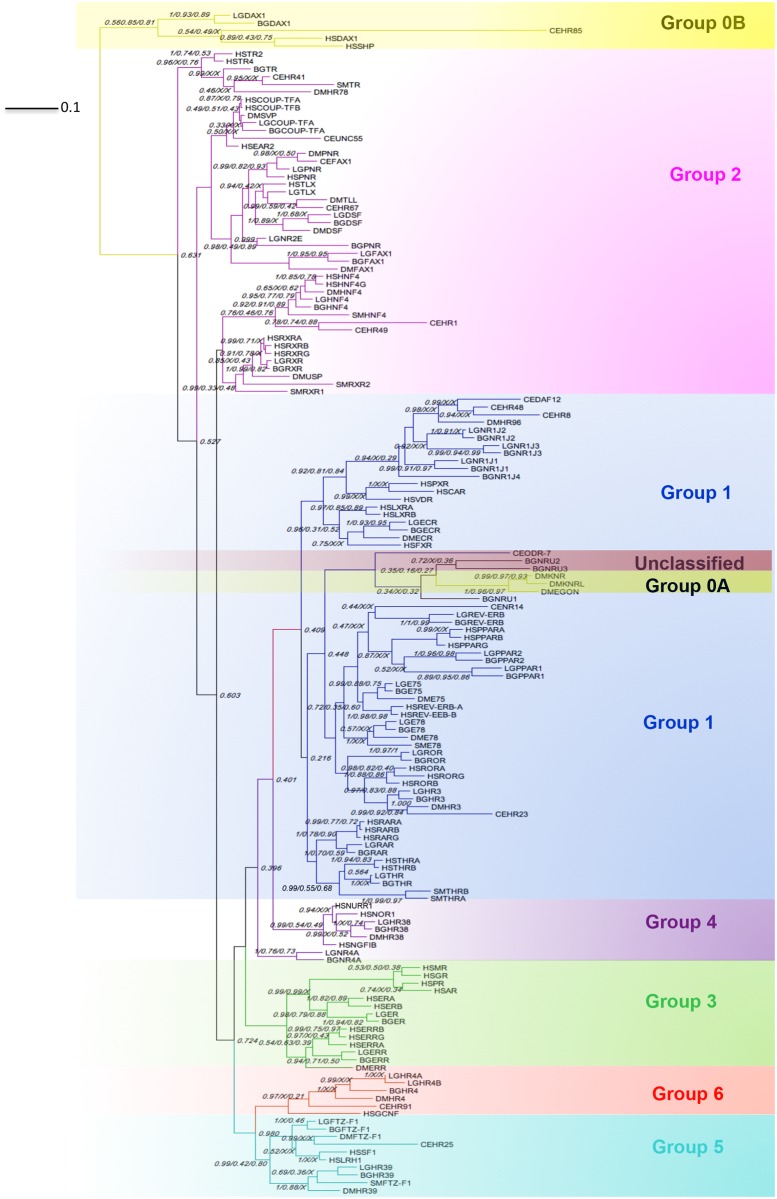
Phylogenetic relationships of NRs in molluscs, humans, fly, nematode and trematode. NRs from six species *B*. *glabrata (Bg)*, *L*. *gigantea (Lg)*, *H*. *sapiens (Hs)*, *D*. *melanogaster (Dm)*, *C*. *elegans (Ce)* and *S*. *mansoni (Sm)*, were subjected to phylogenetic comparisons using Bayesian inference, maximum parsimony and maximum likelihood methods. The Bayesian tree (midpoint rooted) is shown with posterior probability values from Bayesian inference and bootstrap values from maximum parsimony and maximum likelihood trees. The value of 1 on each node represents 100% posterior probability/bootstrap support; an X indicates an area of disagreement from the Bayesian tree (S1 Fig). Scale bar, 0.1 expected changes/site.

The DBD and LBD regions for the full-length molluscan NRs (32 for *B*. *glabrata* and 30 for *L*. *gigantea*) were aligned with *S*. *mansoni*, *D*. *melanogaster*, *C*. *elegans* and *H*. *sapiens* NR sequences (S1 File). Phylogenetic trees were constructed using 3 different approaches: Bayesian Inference (BI), Maximum Parsimony (MP) and Maximum Likelihood (ML) and the resulting trees assessed for concordance. All trees agree at family and subfamily level (S1 Fig, S2 Fig, S3 Fig) but BI shows the greatest resolution at the base of the phylogram and is shown with nodal support generated from all the tree construction methods (Fig 1). The tree shows the main groupings of the NRs.

The position of the unclassified *B*. *glabrata* NRs (BgNRU) is the same in all trees, close to NR0A subfamily within the NR1 group; however the NR0 subfamily was derived to accommodate nuclear receptors that lack either the DBD or the LBD and these 3 *B*. *glabrata* NRs contain both domains (S4 Fig). Therefore these remain as unclassified. The position of members of NR2C/D is also not resolved within Group 2 (Fig 1). Both mollusc genomes each contain one member of this clade that we have designated LgTR/BgTR based on their sequence similarity with HsTR (Table 1). LgTR was not included in the phylogenetic analysis since it was missing the LBD, but BgTR clusters with the other NR2C/D group members Ce_HR41, SmTR and Dm_HR78. The Bayesian tree predicts BgTR as the most distal member of NR2C but the ML and MP trees show both the mollusc sequences to be closely related to DmHR78. However the MP tree placed Ce_HR41 in NR2D, although this NR is usually classified in NR2C. Clearly, the NR2C/D groups seem to be related and not well defined. Within NR1 NR1D was not clearly resolved. In the NR3 subfamily, only the position of the human GR showed non-concordance between the BI tree and the MP tree. All of the trees were in agreement for the NR4/NR5/NR6 subfamilies, with one difference in the position of BgNR4a/LgNR4a and NR5A in the MP tree.

The identified genes have been named in agreement with the unified nomenclature for NRs [23] based on their sequence homology and phylogenetic position. Overall, the identified mollusc NRs encompass almost all the subfamilies, with representatives divided between 21 groups (Table 1) and revealed both similarities and differences in the NR complement between the species used in the study. Seventeen of the 19 subgroups containing human NRs also had representatives in the invertebrate species, including molluscs although in general *H*. *sapiens* contained several NRs where snails had only one, most likely the result of gene or lineage-specific duplication events. The presence of these groups among both protostomes and deuterostomes suggests that these receptors originated in a common ancestor of the bilateria [24,25]. The 2 vertebrate groups not represented in molluscs were the Groups 3C and 1I. Three groups that were common to humans and molluscs, but not found in the other invertebrate organisms included here were 0B (DAX), 1C (PPAR) and 3A (ER). The presence of these receptors in molluscs suggests at least the possibility of commonality in shared signalling pathways, elucidating the evolutionary development of the endocrine system. Seven groups were found only in invertebrates; Groups 1G and 1K only in *C*. *elegans*; 0A only in *D*. *melanogaster*, while groups 1E (E78), 5B (DHR39) 1J and the 2DBD NRs were found in several of the invertebrate species. Representation of these latter groups in *L*. *gigantea* and *B*. *glabrata* shows that these nuclear receptors pre-date the ecdysozoan/lophotrochozoan split.

### Group 3 receptors

The nuclear receptors that bind to steroids, such as androgens, estrogens and progestogens, are responsible for the long-term effects of steroid hormones on reproduction, behaviour, immunity, stress responses and development. The Group 3 steroid receptor family includes the estrogen related receptor (ERR), as well as the estrogen receptor (ER) and group 3C subfamily with the androgen, progesterone, glucocorticoid and mineralocorticoid receptors. Steroid receptors were originally thought to be a vertebrate specific gene family but the identification of the genes with a clear homology to human ERR in trichoplax [73] and an ancestral steroid receptor in amphioxus [74] suggested that these receptors arose early in metazoan evolution and subsequently proliferated in vertebrates through series of gene duplication events [75]. We have identified ER homologues in *B*. *glabrata* and *L*. *gigantea* (Table 1, Group 3A), which have been previously reported in other molluscan species, including bivalves, *C*. *gigas* [37], *Mytilus edulis* [76] and *Chlamys farreri* [77] and gastropods, *Aplysia californica* [38], *Lymnaea ollula* [78], *Marisa cornarietis* [33], *Thais clavigera* [39], *Nucella lapillus* [79], *Bithynia tentaculata* [35] and the cephalopod, *Octopus vulgaris* [36]. The phylogenetic position of the mollusc ER homologue with the vertebrate ER is well supported with a BMCMC posterior probability of 98% and bootstrap values of 0.88 and 0.79 (Fig 1), supporting the suggestion that vertebrate and invertebrate ER diverged from a common ancestor, before the evolution of the deuterostomes [38,75,80]. Structural similarities between ER DBDs of molluscs, annelids, cephalochordates and vertebrates have been documented showing them to bind to and regulate transcription through estrogen response elements [36,40,74,81,82]. The ER LBD in invertebrates may have unique functions, since the mollusc ER homologue, at least *in vitro* (usually in mammalian reporter cell lines), appears to activate transcription constitutively in the absence of a ligand [36,37,39,40] although in annelids the ER has been shown to activate transcription in the presence of estrogens [82]. We also identified a single estrogen-related receptor (ERR) in both *B*. *glabrata* and *L*. *gigantea* (Table 1, Group 3B), which clusters with fly and human ERRs. ERRs have been reported previously in molluscs, including *M*. *cornarietis* [33], *C*. *gigas* (Genbank: EKC20050), *Mizuhopecten yessoensis* (GenBank: BAN84542.1) and *M*. *edulis* [76]. ERRs have no known endogenous ligand, although they are thought to bind to estrogen response elements and may play a role in estrogen signalling and energy metabolism [83].

We identified no convincing homologues in either of the two molluscs for the AR or for any other members of group 3C (Fig 1), which also contains the glucocorticoid receptor (GR), mineralocorticoid receptor (MR) and progesterone receptor (PR). The absence of an AR sequence homologue, both in our systematic exhaustive genomic searches of 2 gastropod species, as well as specific laboratory-based searches in other molluscs [44] using an approach which successfully identified other receptors and another systematic NR survey in the bivalve *C*. *gigas* [45] strongly suggests no vertebrate AR homologue exists in gastropod molluscs. The presence of an AR in molluscs has been hotly contested [84], its existence having been inferred from the effects of androgens and anti-androgens in several species of mollusc (eg. [42,85]), rather than by the identification of a homologous sequence. The absence of an AR means that the findings from previous papers requiring its presence to explain their results may require further analysis in the light of this information; although it should also be noted that the effects of steroids, including those reported for androgens in molluscs may not be mediated via NRs. Steroid hormones have also been shown to act via non-genomic mechanisms in vertebrates, using membrane bound receptors from the G protein family [86–88], although homologues for these receptors have also not yet been identified in molluscs.

The absence of a molluscan AR and the constitutive expression of the ER *in vitro* suggest alternative pathways must exist for spermatogenesis/oogenesis in molluscs and other nuclear receptors have been proposed as initiating these pathways for reproductive processes, some of which exist in vertebrates and which may be particularly important in invertebrates. Although vertebrates have subsequently evolved further processes involving steroid hormones, the presence of orthologous NRs involved in other pathways in molluscs offers the potential opportunity to study these conserved networks in an invertebrate.

### Group 1 and 2 nuclear receptors

**Fig 2:**
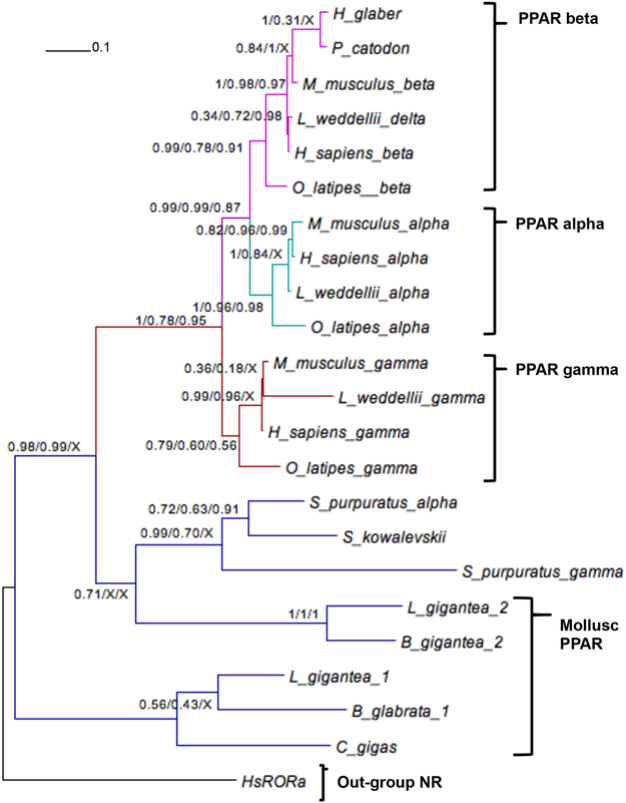
Phylogenetic relationships of peroxisome proliferator-activated receptors (PPAR α, β and γ). PPAR sequences from different species were subjected to phylogenetic analyses using Bayesian inference, maximum likelihood and maximum parsimony. Accession numbers of the PPARs of the different species in the phylogenetic tree: *Heterocephalus glaber* XP_004846774; *Physeter catodon* XP_007109986; *Leptonychotes weddellii* γ XP_006734113; *L*. *weddellii* α XP_006750071; *L*. *weddellii* δ XP_006737129; *Saccoglossus kowalevskii* XP_006819446; *Strongylocentrotus purpuratus* α XP_781750; *S*. *purpuratus* γ XP_784429; *C*. *gigas* EKC18691; *H*. *sapiens* α AB32649; *H*. *sapiens* γ AAH06811; *H*. *sapiens* β AAA36469; *Mus musculus* α NP_035274; *M*. *musculus* γ NP_035276; *M*. *musculus* β NP_035275; *Oryzias latipus* α XP_004069934; *O*. *latipus* β NP_001265836; *O*. *latipus* γ NP_001158348; *B*. *glabrata* 1 Contig2052; *B*. *glabrata* 2 Contig1275; *L*. *gigantea* 1 ProteinID174409; *L*. *gigantea* 2 ProteinID238472; *H*. *sapiens* ROR AAH0883. Scale bar, 0.1 expected changes/site.

Conservation between other NR-mediated pathways in molluscs and humans, even with the absence of Group 3C NRs, still provides the potential to develop a mollusc model for endocrine physiology. The retinoid X receptor (RXR, Group 2B) has been previously identified in molluscs including *B*. *glabrata*, [46], *L*. *gigantea* [25] and *L*. *stagnalis* [89]. We found that the molluscan RXR sequences grouped with good support (BMCMC posterior probability of 91%) close to the human RXR sequences, with the *D*. *melanogaster ultraspiracle* (USP) RXR homologue basal to the clade (Fig 1). BgRXR has been demonstrated to bind and be activated by retinoids, suggesting that retinoid signalling pathways are conserved in the Lophotrochozoa [46]. It was also shown to bind to the response element DR1 either as a homodimer or as a heterodimer with mammalian RARα, LXR, FXR or PPARα [46], which suggests the possibility of conservation of several important signalling pathways in molluscs. The ligand and the co-activator peptide have been previously reported to bind to snail RXR in essentially the same manner as observed in human RXR LBD structures [90], suggesting that the mechanisms of RXR-mediated transcription regulation are very similar in molluscs and humans. The significance of RXR signalling on reproductive physiology in molluscs is strongly supported by the finding that RXR expression is affected during TBT exposure and subsequent development of imposex in *N*. *lapillus* [91]; RXR is thought to be the receptor which mediates TBT induction of imposex in this species [29]. It has also been recently demonstrated that, in conjunction with RXR signalling, peroxisome proliferator-activated receptor (PPAR) pathways are activated by organotins [92] and may induce imposex in response to TBT in *N*. *lapillus* [93]. Convergence of 9-cis retinoic acid, a natural ligand for RXR, and PPAR signalling pathways through PPAR-RXRα hetero-dimerization is well established in mammals [94]. We identified two PPAR homologues in both *L*. *gigantea* and *B*. *glabrata* both containing identical sequence to the P-box of human PPAR (CEGCKGFFRRTI) which grouped with the human PPAR genes (Fig 1); however the 3 human genes seem to have diverged after the split with the molluscan paralogues. Examination of PPAR gene relationships in more detail and including some additional species in the phylogenetic analysis, confirms that the 2 molluscan paralogues diverged prior to the split with vertebrates. The genes we have designated PPAR1 lie basal to the clade, while the vertebrate α, β and γ PPAR genes are orthologues of the gene we have designated PPAR2 (Fig 2).

The RXR also binds with the thyroid hormone receptor (THR, Group 1A). Both gastropods contain a THR homologue (Table 1), which has been previously reported in *L*. *gigantea* and other invertebrates [49]. Wu et al (2007) confirmed that the *S*. *mansoni* THR was able to heterodimerise with the RXR. The molluscan THRs cluster with the human THRα/β (Fig 1). The presence of this NR makes *B*. *glabrata* a potential model for thyroid hormone processes. Thyroid hormones (THs) play key roles in growth development and metabolism in vertebrates. In invertebrates exogenous (food) sourced THs have been suggested to be involved in signalling [95].

*Ultraspiracle* (USP), the fly orthologue of the RXR (reviewed in [96]), has been shown to heterodimerize with the ecdysone receptor (EcR) to control ecdysone signalling in insects driving metamorphosis and moulting [97]. EcR homologues (Group 1H) were identified in both molluscs (Table 1), the first time this has been reported for *B*. *glabrata*. The presence of the EcR ligand, ecdysone, has been previously reported in pulmonate snails, in *B*. *glabrata* and *Lymnaea stagnalis* [98], but its origin (endogenous or exogenous) is still contested [99]. Interestingly, ecdysone exposure was found to affect growth and egg production in *B*. *glabrata* and it has been suggested that ecdysone secreted from the *S*. *mansoni* parasite might provide a mechanism for parasitic castration and gigantism seen in some intermediate hosts when infected [100]. The EcR of *L*. *gigantea* has been previously identified and its 3D structure investigated [101] revealing the ligand-binding pocket of the *L*. *gigantea* EcR homolog has the potential to bind to ecdysone-related steroids. The observation that EcR is expressed in testis tissue may also indicate a role in molluscan reproductive processes [101].

One retinoic acid receptor (RAR) was identified in each gastropod species. RARs have previously been identified in the gastropod *L*. *stagnalis* (GenBank: GU932671), the rock shell, *T*. *clavigera* [102], the bivalve, *C*. *gigas* [45] and recently in *N*. *lapillus* [103]. In vertebrates RAR binds retinoic acid, the biologically active form of vitamin A, which mediates cellular signalling in embryogenic antero-posterior patterning of the central nervous system [104]. However the RAR of *N*. *lapillus* did not bind to all-trans retinoic acid or any other retinoid tested nor was it able to activate the transcription of reporter genes in response to stimulation by retinoids [103]. More work is clearly necessary to elucidate the function of RAR in molluscs.

We also identified homologues of Group 2 C/D vertebrate testicular receptor 4 (TR4) in *B*. *glabrata* and *L*. *gigantea*, (designated as BgTR and LgTR) that in mice have been shown to control spermatogenesis [105] and folliculogenesis [106]. As an orphan nuclear receptor (with no known ligand) the physiological function of TR4 has been difficult to ascertain until the recent development of knock-out mice for this gene (reviewed in [107]). Thus determining the basic biology of vertebrate NRs is dependent on animal experimentation, some of which may be possible in a simplified model system such as a mollusc.

The NR2E nuclear receptors that have been functionally characterized have a common role in nervous system development, for example the *tailless* (*tll*) gene of *D*. *melanogaster* is involved in embryonic CNS and larval eye development [108] and the mouse *Tlx* gene has also been found to be a key component of retinal development and vision [109]. In *B*. *glabrata* we identified homologues of photoreceptor cell-specific nuclear receptor (PNR), *dissatisfaction* (DSF), TLX and FAX1, while in *L*. *gigantea*, we identified a further NR2E gene (Table 1).

Groups NR1J and NR1I cluster in the tree (Fig 1). No members of the NR1I group, including vitamin D receptor (VDR) or pregnane X receptors (PXR), were identified in *B*. *glabrata* and *L*. *gigantea*; however NR1J group in protostomes shares similarity with vertebrate NR1I group [110] and there is evidence that both NR1I/NR1J groups share a common ancestor [24]. NR1I receptors are considered as natural sensors and are involved in xenobiotic metabolism in vertebrates [111]; however, other studies have indicated that NR1J members might regulate xenobiotic responses in *D*. *melanogaster* and *C*. *elegans* [112,113]. Our results show that *L*. *gigantea* has three and *B*. *glabrata* four NR1J representatives (Table 1). The mollusc NR1J group members possess the well-conserved base contact residues (ESCKAFFR) within the DBD, characteristic of the NR1J sub family [114]. In molluscs the NR1J group receptors may be able to perform the same xenobiotic recognition functions as the closely related NR1I of vertebrates.

### Group 5 and 6 nuclear receptors

Germ cell nuclear factor (GCNF) homologues (Group 6) have previously been reported in bilaterians (reviewed in [50]) and we identified them in both gastropod species. This is in contrast to the recently published survey of NRs in the pacific oyster *C*. *gigas* [45] which did not identify any NRs from Group 6. Both BgHR4 and LgHR4a/b cluster with good support (posterior probability 1) to the *D*. *melanogaster* GCNF homologue, Dm_HR4 (Fig 1). During embryonic stages in vertebrates, GCNF can interfere with retinoic acid signalling affecting the expression of *cyp*26*A*1, which is essential for normal hindbrain patterning and early developmental stages [104]. In Group 5 we identified BgFTZ-F1/LgFTZ-F1 and BgHR39/LgHR39, homologues of the *D*. *melanogaster* NR5 subfamily members, *fushi tarazu* factor 1 and hormone receptor 39 respectively, with a conserved stretch of 23 amino acids adjoining the C-terminal end of the zinc finger motif (AVR**S**DRMRGGRNKFG**P**MYKRDRA). This sequence is located immediately after the DBD and plays an important role in high affinity interactions of the receptor with DNA [115]. These Group 5 NRs are related to the vertebrate SF-1 which controls reproductive development and regulates the transcription of steroid-modifying cytochrome P450 genes. In *Drosophila* HR39 is essential for sexual development, required in females both to activate spermathecal secretion and repress male-specific courtship genes, and controls the expression of specific cytochrome P450 genes [116]. The conservation of function between invertebrates and vertebrates in these receptors in Groups NR5 and NR6, suggests the potential for molluscs to be used to model some aspects of mammalian reproductive biology and that further study into the reproductive biology of invertebrates is warranted.

### 2DBD nuclear receptors

**Fig 3:**
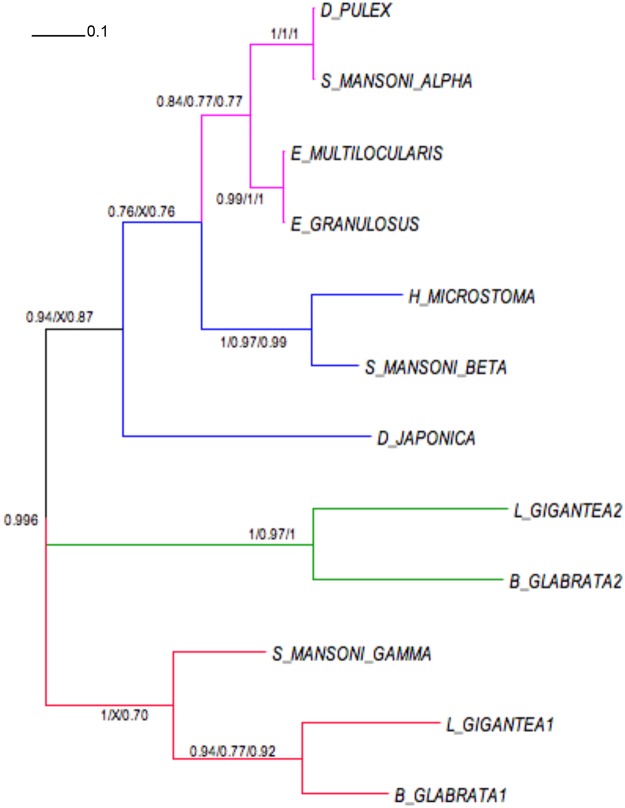
Phylogenetic relationships of 2DBD nuclear receptors (alpha, beta and gamma). 2DBD NR sequences from different species were subjected to phylogenetic analyses using Bayesian inference, maximum likelihood and maximum parsimony. Accession numbers of the 2DBD NRs of the different species in the phylogenetic tree: *D*. *pulex* FE382753; *Hymenolepis microstoma* CDS31978; *Echinococcus multilocularis* CDI99377; *Echinococcus granulosus* CDS24085; *S*. *mansoni* α AAW88533; *S*. *mansoni* β AAW88534; *S*. *mansoni* γ AAW88550; *Dugesia japonica* BP186725; *L*. *gigantea* 2DBD1 ProteinID168696; *L*. *gigantea* 2DBD2 ProteinID173632; *B*. *glabrata* 2DBD1 Contig304; *B*. *glabrata* 2DBD2 Contig1296. Scale bar, 0.1 expected changes/site.

We identified two 2DBD-NRs in *B*. *glabrata* as well as the two 2DBD-NRs previously reported from *L*. *gigantea* [48]. We compared these to the 3 previously identified from *S*. *mansoni* [71] as well as 2DBD NRs from other organisms (Fig 3). The gastropod 2DBD1 clusters with Sm2DBDγ while the position of the second gastropod 2DBD was not determined within the clade. Both the identified Bg 2DBD-NRs possess the two tandem DNA binding domains, and a well-conserved P-box sequence (CEACKK) in the first DBD and a P-box (CEGCK) in the second DBD. The P-box of the second DBD is similar to DBD of NR1 family but the P-box of the first DBD is unique to this NR and may determine novel targets [48].

### NRs in parasite and intermediate host

The discovery of differences and similarities in the nuclear receptor repertoire of snail host and *S*. *mansoni* may open up avenues to further characterize host-parasite interactions and potentially to interfere with schistosome development within the host. Parasites are known to interfere with the snail reproductive system, since one effect of trematode infection in snails is parasitic castration (reviewed in [117]). The intimate host-parasite and intermediate host-parasite relationships, makes a comparison of nuclear receptors in all three organisms significant. It has been suggested, at least for the definitive host, that the ability of the parasite to exploit the hosts’ hormonal microenvironment may be critical to allow it to establish, grow and reproduce [118]. This may also be significant in enabling the parasite to inhabit its intermediate host and may depend upon shared nuclear receptors. All 3 organisms have one or more THR, RAR, HNF4, RXR, TR, COUP-TF and similar NRs in Groups 2E, 4A and 5A. Only snails and schistosomes have the E78, HR39, HR96 and 2DBD NRs, while snails and humans only have DAX1, PPAR, Rev-erb, ROR, ER, ERR and group 6A NRs. NRs which are specific to the snails (in this three-way comparison) are the EcR and the group of unclassified NRs, designated as BgNRU1, BgNRU2, BgNRU3, and BgNRU4 respectively and placed in a separate group (Table 1). These unclassified receptors may have originated from a specific duplication event in a *B*. *glabrata* precursor, or alternatively, they could be remnants of ancient NR subfamilies, whose representatives have been secondarily lost in the other represented species. With regard to the EcR, given that snails have a EcR homologue and schistosomes do not, the suggestion that β-ecdysterone acts as an attractant in host location by miracidia and affects the rate of growth and maturation in snails [100] is potentially interesting, although the ability of snails to make ecdysone is not proven [99]. Both organisms have homologues of the *D*. *melanogaster* NRs E78 and HR39 which are active in ecdysone signalling [116,119].

In addition to discovering the intricacies of the molecular interactions between host and parasite with a view to disrupting the development of the schistosome, the other key strategy for schistosomiasis control is to reduce the numbers and/or distribution of the intermediate host snails. Currently molluscicides, such as the commercially available niclosamide-based Bayluscide, are effectively employed in schistosomiasis control programs; however the problems of resistance and toxicity to other organisms means that the search for alternative, more selective, compounds is on-going. The capacity of nuclear receptors to bind small ligands, including exogenous substances such as natural products and synthetic chemicals, makes them potential targets for molluscicides [120].

### *Biomphalaria glabrata* as a model organism

There is a long tradition in biology of examining biochemical processes in simplified models to elucidate mechanisms in more complex organisms. We have demonstrated that there are some NR groups with a single representative in the mollusc species’ examined instead of the multiple genes from humans. For example in the NR1 subfamily, both molluscs have single homologues of several significant NRs, such as the retinoic acid receptor (RAR), THR and ROR. For these groups, *B*. *glabrata* could provide a simple model system, not only to study the development and diversification of endocrine systems, but also to investigate and test gene function and response to external stimuli. The identification of many vertebrate-like NRs in *B*. *glabrata* could make it a suitable model candidate to investigate functional relationships of individual receptors. The ultimate choice of model organisms lies not only with the biology of the organism, but also on its tractability for study and manipulation. Aside from the genome, the full range of genomic tools available for *B*. *glabrata*, such as RNAi [8,121], BAC library [5] and microarrays [6,122,123] as well as the potential to use classical approaches of enhancer and suppressor genetics and transgenics to explore regulatory networks, make *B*. *glabrata* a good candidate. However, the advent of next generation sequencing makes transcriptome study from non-model organisms a real possibility and therefore other mollusc species may ultimately make better model systems. In particular, this approach could be used to identify inputs from signal transduction pathways, potential hormone metabolic genes, co-activators, co-repressors and other unknown factors that may impinge upon receptor activity. Based on the absence of Group3C NRs, it can be concluded that *B*. *glabrata* is an inappropriate model for mammalian steroid hormone function mediated via NRs, as the genes for several major steroid hormone receptors are not present. However since steroid hormones have also been shown to act via non-genomic mechanisms in vertebrates, using membrane bound receptors (mPR and GPR30) [87] there is still potential to examine alternative pathways in these organisms.

## Conclusions

We identified 39 nuclear receptor genes in *B*. *glabrata* and 33 in *L*. *gigantea* representing all seven principal vertebrate nuclear receptor groups. Molluscan endocrinology is poorly understood, so further study to determine the functionality of these identified NRs promises new insights, especially concerning the many unanswered questions regarding the effects of steroids and other EDCs on molluscs. The importance of the snail as an intermediate host for schistosomes, also justifies further investigation into the function of such genes. The absence of vertebrate NRs such as VDR, CAR and PXR, as well as steroid hormone NRs, AR/PR/GR/MR for which we found no orthologues in the two mollusc species examined in this study, indicates that several significant signalling pathways are absent in gastropods. Nevertheless, we have identified an array of NRs common to both vertebrates/molluscs and molluscs/flies. These results add weight to Thummel’s speculation about the convergent regulation of NRs in vertebrates and invertebrates [124]. Elucidation of NR targets in molluscs may unlock their potential as new model organisms allowing the discovery of new pathways leading to similar phenotypes found in vertebrates or, indeed, similar pathways that produce a different phenotype; both of which could potentially form simple test assays, for determining gene function or drug/chemical testing. The range of phenotypes, and their underlying genetic mechanisms, available for study in different species may enable the identification of alternative pathways mediated by NRs that might also be exploited [125]. The potential of many invertebrate species for endocrine study is yet to be explored, but the underlying fundamental similarities and differences between molluscs and vertebrates may be the solution to determining not only the endocrine mechanisms of molluscs but also the full intricacies of our own.

## Supporting Information

S1 Fig Bayesian tree showing nuclear receptors in molluscs, human, fly, nematode and trematode. The NRs from six different species were subjected to phylogenetic comparisons using Bayesian inference. The Bayesian tree (midpoint rooted) with posterior probability values is shown. Notations Bg, Lg, Hs, Dm, Ce and Sm in association with receptor names denote sequences from *B*. *glabrata*, *L*. *gigantea*, *H*. *sapiens*, *D*. *melanogaster*, *C*. *elegans* and *S*. *mansoni* respectively.
(PDF)

S2 Fig Maximum Likelihood tree showing nuclear receptors in molluscs, human, fly, nematode and trematode. The nuclear receptors from six different species were subjected to phylogenetic comparison using maximum likelihood method using Jones-Taylor-Thornton (JTT) substitution model. Node labels indicate bootstrap values. Notations Bg, Lg, Hs, Dm, Sm and Ce in association with the receptor name denote sequences from *B*. *glabrata*, *L*. *gigantea*, *H*. *sapiens*, *D*. *melanogaster*, *S*, *mansoni* and *C*. *elegans*, respectively.
(PDF)

S3 Fig Maximum Parsimony tree showing nuclear receptors in molluscs, human, fly, nematode and trematode. The nuclear receptors from six different species were subjected to phylogenetic comparison using maximum parsimony. Node labels indicate bootstrap values. Notations Bg, Lg, Hs, Dm, Sm and Ce in association with the receptor name denote sequences from *B*. *glabrata*, *L*. *gigantea*, *H*. *sapiens*, *D*. *melanogaster*, *S*. *mansoni* and *C*. *elegans*, respectively.
(PDF)

S4 Fig Sequence alignment file showing the unclassified NRs from *B*. *glabrata* as different genes with overlapping regions (DBD and LBD). (PDF)

S1 File A clustal alignment file of the conserved domain regions (DBD and LBD) of all the nuclear receptors included in the study. Notations Bg, Lg, Hs, Dm, Ce and Sm in association with receptor names denote sequences from *B*. *glabrata*, *L*. *gigantea*, *H*. *sapiens*, *D*. *melanogaster*, *C*. *elegans*, and *S*. *mansoni* respectively.
(TXT)

S1 Table Oligonucleotide primers used in RT-PCR analyses of nuclear receptor genes. Forward and reverse primer sequences with their product size (as submitted to GenBank), their corresponding GenBank accession numbers and their optimised annealing temperatures.
(DOCX)

S2 Table *L*. *gigantea* transcripts. Detailed summary of all the top BLAST expressed sequence tag (EST) hits for *L*. *gigantea* NRs, including the EST description, GenBank accession number and E-value.
(PDF)
