# Supplementary figures and images for: The Nuclear Receptors of Biomphalaria glabrata and Lottia gigantea: Implications for Developing New Model Organisms
Source: PLoS One. 2015 Apr 7;10(4):e0121259. doi: 10.1371/journal.pone.0121259 (PMC4388693; doi:10.1371/journal.pone.0121259)

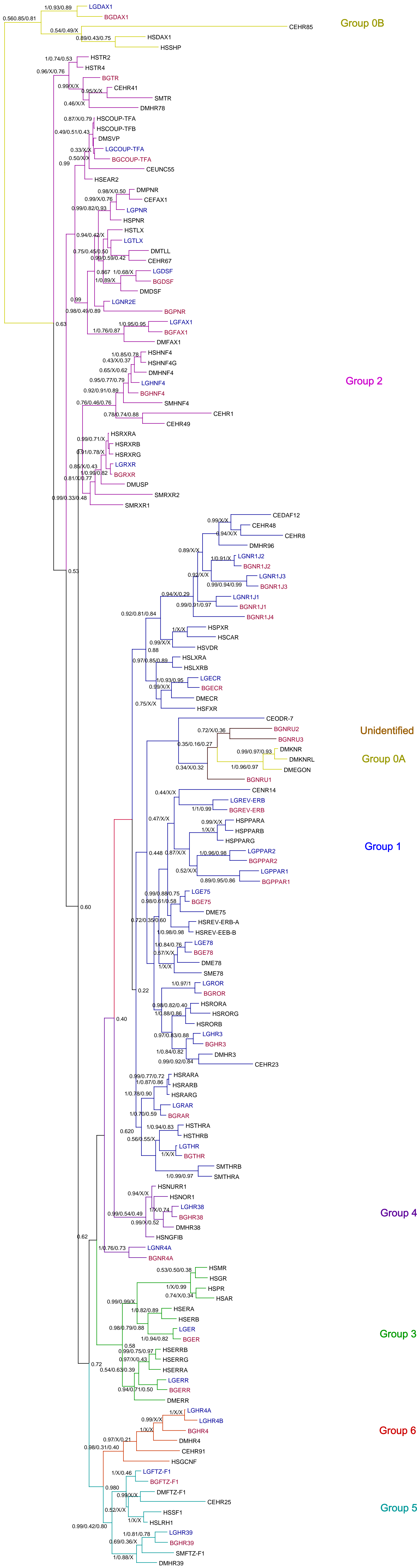

Supplement: S1 Fig — The NRs from six different species were subjected to phylogenetic comparisons using Bayesian inference. The Bayesian tree (midpoint rooted) with posterior probability values is shown. Notations Bg, Lg, Hs, Dm, Ce and Sm in association with receptor names denote sequences from B. glabrata, L. gigantea, H. sapiens, D. melanogaster, C. elegans and S. mansoni respectively. (PDF) [file pone.0121259.s001.pdf]

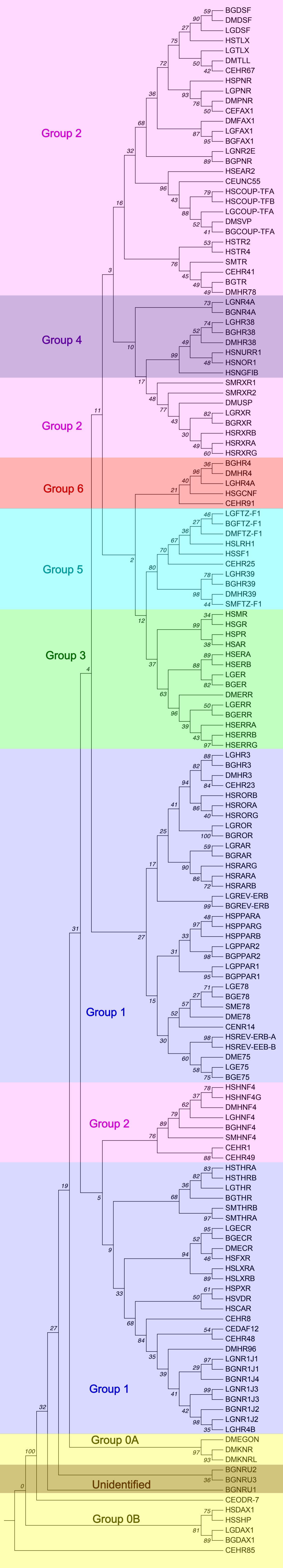

Supplement: S2 Fig — The nuclear receptors from six different species were subjected to phylogenetic comparison using maximum likelihood method using Jones-Taylor-Thornton (JTT) substitution model. Node labels indicate bootstrap values. Notations Bg, Lg, Hs, Dm, Sm and Ce in association with the receptor name denote sequences from B. glabrata, L. gigantea, H. sapiens, D. melanogaster, S, mansoni and C. elegans, respectively. (PDF) [file pone.0121259.s002.pdf]

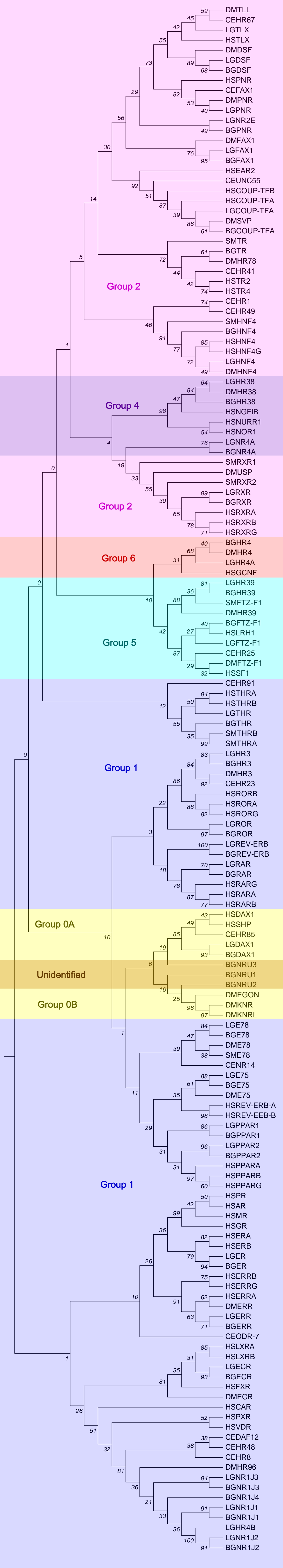

Supplement: S3 Fig — The nuclear receptors from six different species were subjected to phylogenetic comparison using maximum parsimony. Node labels indicate bootstrap values. Notations Bg, Lg, Hs, Dm, Sm and Ce in association with the receptor name denote sequences from B. glabrata, L. gigantea, H. sapiens, D. melanogaster, S. mansoni and C. elegans, respectively. (PDF) [file pone.0121259.s003.pdf]
