# Supplementary material for: The Nuclear Receptors of Biomphalaria glabrata and Lottia gigantea: Implications for Developing New Model Organisms
Source: PLoS One. 2015 Apr 7;10(4):e0121259. doi: 10.1371/journal.pone.0121259 (PMC4388693; doi:10.1371/journal.pone.0121259)
Supplement: S4 Fig — (PDF) [file pone.0121259.s004.pdf]

```
              [DBD region]
BGNRU3     LPPCKICGEP ASGFHYGLNS CGACKGFFLR SLLRPDPYKC STDGKCQVGP
BGNRU1     LPPCRVCGNK ASGLHFGVNT CEACNEFFRR SLKRGASYYC TKNRECQVYG
BGNRU4     LPPCRVCGDD GAGFHYGVNT CEACKGFFHR SLKVHHLYKC SGSGSCDLTK
BGNRU2     LPPCRVCDAP GAGFHYGANT CEACKGFFHR SLKLHEQYRC DGEGHCTIE.

                                          [LBD region]
BGNRU3     .EIRKICQKC RFDKCLRLAI KIGLGIHNEI ATVIKFFKQI PGFKHLPTSD
BGNRU1     .KKRNACSYC RYRRCVEMGM SRDEMTEKWL RNYINYAKNV PGFKELALSD
BGNRU4     IKRQRSCQYC RYQRCVQVGM SKG....... .......... ..........
BGNRU2     .HGLKLCQLC RYQKCLSVGM GKDIWLENMI RGMVKFAKAI PGFGKVITPD

BGNRU3     QIKLVKGCSH EYHILGLYRG WDNTTRCMTF ATETLTEQDL RKVFP....D
BGNRU1     QASLVRGTWF EFWFLGAYRG YNSDLRVVYY PNRTFHEEEI IKVFG....K
BGNRU4     .......... .......... .......... .......... ..........
BGNRU2     KVNLIKYSRQ EFCVFSIYTT LNCELGVMRG GDEWKCEYDI AKAGSFTAFK

BGNRU3     EALSAHIQMV MTLQKLNLSS EQIVLLKAIV ATAPDRDILE TVEAVTHIHW
BGNRU1     EYTDFSFSLA DRMASLRVTP EEMVLIKTVC LTFPDRTPLQ DRDAVESMHW
BGNRU4     .......... .......... .......... .......... ..........
BGNRU2     EFIEKNFNFC KTLQNLKLTE QEQVVIKAIL IMAPDRFPPV ESSLAHEIYW

BGNRU3     KLVNCLLYLL CKQDLCPVIK FGQIMSVITE MRSYS
BGNRU1     QMVSCLLHTL EKNRPGDSTV FPMVVNKLVE LRXXX
BGNRU4     .......... .......... .......... .....
BGNRU2     YLIKCLLHLL SLNDPKPNLK FAKIISRLTE ARTMT
```
