# Supplementary material for: The Nuclear Receptors of Biomphalaria glabrata and Lottia gigantea: Implications for Developing New Model Organisms
Source: PLoS One. 2015 Apr 7;10(4):e0121259. doi: 10.1371/journal.pone.0121259 (PMC4388693; doi:10.1371/journal.pone.0121259)
Supplement: S1 Table — Forward and reverse primer sequences with their product size (as submitted to GenBank), their corresponding GenBank accession numbers and their optimised annealing temperatures. (DOCX) [file pone.0121259.s006.docx]

| **Putative NRs** | **Forward primer** | **Reverse primer** | **Genbank Acc.** | **Fragment size (bps)** | **Annealing temperature** |
| --- | --- | --- | --- | --- | --- |
| BGCOUPTFA | GTCCGAGAGCAACAACAACA | CTGACGAGAACAGAGCGATG | JZ390916 | 773 | 63^0^C |
| BGNRU1 | TGAGTTTTTCAGGCGGAGTT | GCAGCAGACAAGACACCATC | JZ390900 | 867 | 58^0^C |
| BGHR38 | CACCGCACAATAGGTCTCC | TTGTAGGTGCAGTGGTCTCG | JZ390932 | 816 | 55^0^C |
| BGHR3 | AGCAGCCTTCAGTTCTCACC | CAGGGCGATTGAGATTGG | JZ390936 | 913 | 58^0^C |
| BGPPAR2DBD | GGTGACAAAGCGTCTGGATT | CTTTTTGGCAAGGGTGGTAA | JZ390908 | 492 | 55^0^C |
| BGPPAR2LBD | AAGAGTGCCAGCCATTTAGC | CATTGCCAGTTCTGTTTCCA | JZ390909 | 469 | 56.7^0^C |
| BGHNF4 | CAAGTTGGCGACTCTGTGAA | GGCTGTTGAGGACTGGGTTA | JZ390914 | 914 | 63^0^C |
| BGER | CACCACCCCTGAATCTGTCT | ACTTGCCGCATCTCTTGAAT | JZ390913 | 1097 | 55^0^C |
| BGERR | GATCCTTCCCACGACGACTA | TCAGCATCCAACATTTCCAA | JZ390912 | 1033 | 55^0^C |
| BGROR | CAGCCCTACCCTCAACACTC | CTATGGCAACAGGTCAAAG | JZ390933 | 879 | 58^0^C |
| BGECR | AGCAGTGCTTACCACCCATT | AGTCCAGGACGCTCTGAAAA | JZ390899 | 999 | 55^0^C |
| BGFAX1 | GCAAGCACTACGGGGTCTAC | TCAACACGCTTTGCCTCTAA | JZ390935 | 731 | 58^0^C |
| BGHR4DBD | CGTCCTCTTCTGGGGTGATA | CTGGGATTGTCTCTGGCATT | JZ390920 | 779 | 55^0^C |
| BGHR4I | GGATGGTTCTTGCAGCTG | GTCGATGCGTTCAACTTC | JZ390921 | 833 | 55^0^C |
| BGHR4LBD | CCCTCCAACTCATCAAGCAT | CTGAGAAGGTGGGCCTGTAG | JZ390922 | 372 | 55^0^C |
| BGNR1D1 | TGTCAACCTAACCCGTGGAT | GGAAGTGGGCTGAGAACATC | JZ390906 | 938 | 55^0^C |
| BGNR1D2 | GCTGTAAGGCTTGTCGGTTC | GACTCGGTTGGCTTCTGTTC | JZ390907 | 1070 | 63^0^C |
| BGNR1D3 | AACCCCACTGAACGAGACAC | GGGCTCCATCCTTTTTGAA | JZ390902 | 814 | 55^0^C |
| BGNR1J3DBD | GAGACTCGGAGCACAACACA | GATCGGCCAGCTTCTCTATG | JZ390897 | 855 | 58^0^C |
| BGNR1J3LBD | CAGTGGAGAGACAGCAACGA | CAGCAACATCTTGGAGTGGA | JZ390898 | 661 | 55^0^C |
| BGTRDBD | GCCTCTGTGGAAACCAAAAA | CCCCGACAAGCATAACCTAA | JZ390926 | 376 | 55^0^C |
| BGTRI | GAACGTCGTCCAAGTGAC | GTCTGGAGGTCAGCATTG | JZ390927 | 701 | 55^0^C |
| BGTRLBD | CGTTCAGTCACAGCCTTTCA | ACCAGTAATCCCAGCCACAG | JZ390928 | 608 | 55^0^C |
| BGNR4A | GACAGCATCACCCCAGAGTT | ACGGCAGTCGCAAAAGTATC | JZ390905 | 928 | 60.6^0^C |
| BGNRU2DBD | AATGGGCCGAAAAAGAAAAC | AGAAGGGGTAAGCGGTGAAT | JZ390903 | 805 | 63^0^C |
| BGNRU2LBD | ATTCACCGCTTACCCCTTCT | GCTAGGCTGGACTCAACTGG | JZ390904 | 894 | 63^0^C |
| BG2DBDNR | CCTCCAATCATCCATTCCAC | CTCCAGTCGGGGCTTTTATC | JZ390896 | 737 | 63^0^C |
| BGTLX | CGGAATATGGCGGAGTTTTA | CACGGAGGGATGGTAGAAGA | JZ390910 | 699 | 58^0^C |
| BGRXR | ATCTGTGCAATATGCGGTG | AAATGTGTCAATGGGCTGGT | JZ390931 | 1059 | 60.6^0^C |
| BGRAR | TTCCTGTGAGGGCTGTAAG | GCTGGACAATGATGGTGATG | JZ390929 | 1030 | 63^0^C |
| BGE78 | CTTTCGACGCAGCATACAGA | CGGAGTTCAGGCAGTTTCAT | JZ390894 | 905 | 63^0^C |
| BGHR39 | CCCTGTGTGTGGGGATAAAG | CTGGAAGCCTCAAGAGCAAG | JZ390901 | 772 | 63^0^C |
| BGNRU3DBD | GGCAACAAAACCAAACGACT | TTTCACGAGGACATTCACCA | JZ390923 | 379 | 63^0^C |
| BGNRU3I | CTGCCCGATTCTATTGAG | CTTCGGCATTGGCTTTG | JZ390924 | 633 | 56.7^0^C |
| BGNRU3LBD | TGCACCAAGACACCAGAGAG | TGTTGGAGTAGGAAGCACGA | JZ390925 | 908 | 63^0^C |
| BGPPAR1 | CGTCCGACAGTGGCAAACA | TGGCATGACATGGCTGAAAT | JZ390930 | 1074 | 63^0^C |
| BGNR1J2 | ACAACTTTGACGCCATCTCC | CAACTCAATGCTCCCACCTT | JZ390911 | 582 | 58^0^C |
| BGNR1J4 | GAAAACAGAGCAAAACGTGAGC | CATTAGGGGTTCCACATCCA | JZ390915 | 862 | 58^0^C |
| BGNR1J1 | CAATCCAAGCGTGAACAAAAG | GGGGAGGCAGTGTCAAAGTA | JZ390895 | 509 | 58^0^C |
| BGFTZ-F1DBD | CTTACCGACGGAAACTCTGC | CCCATTCCACCATCAAAAAC | JZ390917 | 573 | 55^0^C |
| BGFTZ-F1I | GCCACCTGGCTCTCCTG | CAGACACTGTGGACATTC | JZ390918 | 907 | 55^0^C |
| BGFTZ-F1LBD | GTTTTTGATGGTGGAATGGG | ACTTGTCCGTCATCTCTGGG | JZ390919 | 415 | 55^0^C |
| BGDSF | CCTCAAGTCACCGGAAAGA | AATGAAGATGGATGGCTGC | JZ390934 | 518 | 58^0^C |
| BGE75 | GTCTTATGCAGGGTGTGTG | CCTTGTGGTTGGTGTTGATG | JZ390939 | 945 | 63^0^C |
| BGREV_ERBDBD | GTGACGAGTCCTCCGGTTTT | TGAAGGCTGTGTGGTTGTTG | JZ390937 | 367 | 58^0^C |
| BGREV_ERBLBD | CCTCTTCAACCACACAGCCT | GACTAAAGCGAGCCACCATC | JZ390938 | 560 | 63^0^C |
