# Supplementary material for: The Nuclear Receptors of Biomphalaria glabrata and Lottia gigantea: Implications for Developing New Model Organisms
Source: PLoS One. 2015 Apr 7;10(4):e0121259. doi: 10.1371/journal.pone.0121259 (PMC4388693; doi:10.1371/journal.pone.0121259)
Supplement: S2 Table — Detailed summary of all the top BLAST expressed sequence tag (EST) hits for L. gigantea NRs, including the EST description, GenBank accession number and E-value. (PDF) [file pone.0121259.s007.pdf]

Additional file 3

Sequence Name	Sequence Description	E value	Hit Accession
LgDAX1	caxs lottia gigantea from radula and heart lottia gigantea cdna clone caxs4873 5 mrna	0	FC610206
LgTHR	caxu lottia gigantea from female gonad lottia gigantea cdna clone caxu8659 5 mrna	0	FC646817
LgRAR	cbgc lottia gigantea 15h 18h embryos lottia gigantea cdna clone cbgc7034 5 mrna	0	FC805285
LgPPAR1	NA		
LgPPAR2	cbbn lottia gigantea larvae lottia gigantea cdna clone cbbn9244 5 mrna	0	FC775242
LgE75	NA		
LgRev_erb	cbbn lottia gigantea larvae lottia gigantea cdna clone cbbn8629 5 mrna	0	FC774887
LgE78C	caxw lottia gigantea from female gonad lottia gigantea cdna clone caxw9550 5 mrna	0	FC673655
LgHR3	cbbn lottia gigantea larvae lottia gigantea cdna clone cbbn11308 5 mrna	0	FC759257
LgROR	caxx lottia gigantea from male gonad lottia gigantea cdna clone caxx3392 5 mrna	0	FC692720
LgEcR	caxx lottia gigantea from male gonad lottia gigantea cdna clone caxx9325 5 mrna	0	FC701413
LgNR1J1	NA		
LgNR1J2	caxp lottia gigantea from radula and heart lottia gigantea cdna clone caxp4376 5 mrna	0	FC587866
LgNR1J3	NA		
LgHNF4	caxp lottia gigantea from radula and heart lottia gigantea cdna clone caxp2428 5 mrna	0	FC584194
LgRXR	cbbn lottia gigantea larvae lottia gigantea cdna clone cbbn11953 5 mrna	0	FC760240
LgTR	caxp lottia gigantea from radula and heart lottia gigantea cdna clone caxp1708 5 mrna	0	FC582901
LgTLX	NA		
LgDSF	NA		
LgFAX1	NA		
LgNR2E	caxp lottia gigantea from radula and heart lottia gigantea cdna clone caxp7152 5 mrna	0	FC592666
LgPNR	NA		
LgCOUP-TFa	cbbn lottia gigantea larvae lottia gigantea cdna clone cbbn11647 5 mrna	0	FC759752
LgER	caxp lottia gigantea from radula and heart lottia gigantea cdna clone caxp6663 5 mrna	0	FC591742
LgERR	cbgc lottia gigantea 15h 18h embryos lottia gigantea cdna clone cbgc18627 5 mrna	0	FC788262
LgHR38	NA		
LgNR4a	caxx lottia gigantea from male gonad lottia gigantea cdna clone caxx755 5 mrna	0	FC699271
LgFTZ-F1	CBBN9480.fwd CBBN Lottia gigantea 3,4,5,6.5d Larvae (M) Lottia gigantea cDNA clone CBBN9480 5-, mRNA sequence	0	FC775593
LgHR4a	caxp lottia gigantea from radula and heart lottia gigantea cdna clone caxp4376 5 mrna	0	FC587866
LgHR4b	NA		
LgHR39	NA		
Lg2DBDNR1	caxx lottia gigantea from male gonad lottia gigantea cdna clone caxx20789 5 mrna	0	FC689646
Lg2DBDNR2	NA		
